# Associations between Childhood Body Size, Composition, Blood Pressure and Adult Cardiac Structure: The Fels Longitudinal Study

**DOI:** 10.1371/journal.pone.0106333

**Published:** 2014-09-05

**Authors:** Roy T. Sabo, Miao-Shan Yen, Stephen Daniels, Shumei S. Sun

**Affiliations:** 1 Department of Biostatistics, School of Medicine, Virginia Commonwealth University, Richmond, Virginia, United States of America; 2 Department of Pediatrics, School of Medicine, University of Colorado, Aurora, Colorado, United States of America; University of Bologna, Italy

## Abstract

**Objectives:**

To determine whether childhood body size, composition and blood pressure are associated with adult cardiac structure by estimating childhood “age of divergence.”

**Methods:**

385 female and 312 male participants in the Fels Longitudinal Study had echocardiographic measurements of left ventricular mass, relative wall thickness, and interventricular septal thickness. Also available were anthropometric measurements of body mass index, waist circumference, percentage body fat, fat free mass, total body fat, and systolic and diastolic blood pressures, taken in both childhood and adulthood. The age of divergence is estimated as the lowest age at which childhood measurements are significantly different between patients with low and high measurements of adult cardiac structure.

**Results:**

Childhood body mass index is significantly associated with adult left ventricular mass (indexed by height) in men and women (ages of divergence: 7.5 years and 11.5 years, respectively), and with adult interventricular septal thickness in boys (age of divergence: 9 years). Childhood waist circumference indexed by height is associated with left ventricular mass (indexed by height) in boys (age of divergence: 8 years). Cardiac structure was in general not associated with childhood body composition and blood pressure.

**Conclusions:**

Though results are affected by adult body size, composition and blood pressure, some aspects of adult cardiac structure may have their genesis in childhood body size.

## Introduction

A recent review of echocardiographic studies shows that the incidence of left ventricular hypertrophy (LVH) remains high in most races and both genders despite advances in hypertension management over the past two decades.[1] This high LVH incidence has coincided with an increase in the prevalence of childhood obesity over the previous three decades in the United States,[2] across all gender, race and socioeconomic groups.[3] Several studies have found associations between obesity,[4]–[10] body size,[11]–[13] adiposity[14], [15], and blood pressure[10], [16], [17] and left ventricular mass (LVM), mass index (LVMI), or hypertrophy (LVH), while others have linked obesity and adiposity to relative wall thickness (RWT)[18] and other measures of cardiac structure.[14], [15] LVM has also been shown to independently predict the incidence of several clinical events – including fatality – attributable to cardiovascular disease.[19]

The ability to predict cardiac structure in adults by measuring body size, composition and blood pressure in childhood is of clinical and public health importance, as such predictions might suggest corrective interventions that can be implemented in early childhood or adolescence. While some studies have linked childhood body size measurements with certain cardiovascular outcomes (such as mortality and hypertension), [20], [21] fewer studies have focused on associations between childhood body size and adulthood cardiac structure. Notably, as part of the Bogalusa Heart Study (BHS), Toprak *et al*. found that childhood body mass index (BMI) was a significant factor in determining eccentric left ventricular hypertrophy in adulthood. [22] Urbina *et al*. – also from the BHS – similarly found associations between childhood body size and LVMI in young adults. [23]

In this manuscript we focus on associations between childhood body size, composition and blood pressure with adult cardiac structure. Using longitudinal data from the Fels Longitudinal Study, we estimate “ages of divergence” in childhood growth trends of BMI, waist circumference (WC), percentage body fat (%BF), fat free mass (FFM), total body fat (TBF), systolic (SBP) and diastolic (DBP) blood pressure, based on adult measures of LVMI, RTW, and interventricular septal wall thickness (IVST). We define age of divergence as the age at which the difference in a particular childhood body size, composition or blood pressure measurement (between participants with low and high adult cardiac structure values) becomes significant and generally remains significant throughout the remainder of the growth trajectory [21]. For our purposes, we define high and low as the third and first quartiles, respectively, of the adult echocardiographic measurement in question. We also adjust (in separate analyses) for adult body size, composition and blood pressure, as well adult lifestyle measurements, such as alcohol use, smoking status, and level of physical activity.

## Methods and Procedures

### Ethics Statement

All participants provided written informed consent to participate in this study, and all procedures were approved by the Institutional Review Boards at Wright State University and Virginia Commonwealth University.

### Participants

This study examined a non-random subsample of European-American male and female participants of the Fels Longitudinal Study (FLS) who were selected to undergo a echocardiographic examination. All FLS participants who were at least 20 years old and – for females – were not pregnant were approached during their routine FLS visits between 12/1/1999 and the end of data collection on 6/30/2009 to participate. Out of a total of 1,215 active FLS participants, 471 females and 405 males agreed to undergo echocardiographic measurement. Among those, 385 females and 312 males were greater than 20 years of age. Among those participants meeting the selection criteria: seven men and seven women had been diagnosed with cardiovascular disease (including stroke and heart attack); seven men and three women had type 2 diabetes mellitus (with fasting glucose exceeding 125 mg/dL); six men and two women were treated with insulin; three men and one woman had chronic obstructive pulmonary disease; 16 men and 18 women had been prescribed antihypertensive medication; and no participants had known congenital heart disease. These participants and their measurements were not removed from the study sample.

The FLS has enrolled participants continuously since 1929. Participants are generally enrolled at birth and are not selected in regard to factors known to be associated with disease, body composition or other clinical conditions. FLS subjects are examined semi-annually until 18 years of age, and biennially thereafter. Two textbooks contain more detailed information on the FLS beyond the sub-sample covered here. [24], [25]

### Measurements

The echocardiographic measurements were performed by a certified sonographer under the supervision of Dr. Stephen Daniels, using an ATL Philips Medical System HDI 5000 ultrasound imaging system. Two-dimensional and two-dimensional directed M-mode echocardiographic images were recorded, and measurements were made on three or more cardiac cycles according to the recommendations of the American Society of Echocardiography (ASE). [26] Left ventricular mass was calculated using the ASE formula: LVM = 0.8(1.04 ([LVIDd+PWTd+IVSTd]^3^-[LVIDd]^3^))+0.6 g, where LVIDd is left ventricular internal dimension at end diastole, PWTd is posterior wall thickness at end diastole, and IVSTd is interventricular septal wall thickness at end diastole. Relative wall thickness was calculated as: RWT = 2(PWTd)/(LVIDd). Interventricular septal wall thickness at systole (IVSTs) was also recorded. Since LVM is height-dependent, we divided LVM by height raised to the 2.7 power (LVMI) as previously suggested. [26], [27]

Anthropometric body size measurements were taken following recommendations in the *Anthropometric Standardization Reference Manual*. [28] Weight was measured to 0.1 kg using a SECA scale. Height was measured to 0.1 cm using a Holtain stadiometer. BMI was then calculated as the ratio of weight to height (in meters) squared (kg/m^2^). WC was measured at the level of the highest point on the right iliac crest in a plane parallel with the floor. Since WC is dependent upon height, [29] we indexed WC by dividing by height (WCHt). All measurements were taken twice, and a third measurement was taken if the difference between the first two exceeds an established tolerance (0.3 kg for weight, 0.5 cm for height, and 0.1 cm for waist circumference), and the average values were used for analysis.

Body composition measurements of FFM and TBF were made by a Lunar LPX and a DXA Hologic QDR 4500 Elite densitometer (Hologic, Waltham, MA). The coefficient of variation (CV) is 3.5**%** for soft tissue. Our DXA procedures have been compared and calibrated with those of underwater weighing (uww) [30], which is important in this study as body composition measurements for some older participants were taken using uww. To obtain consist body composition measurements across all participants, the following conversion equations were used: TBFuww = 2.1582+(1.1533xTBFdxa); FFMuww = 1.8449+(0.9329xFFMdxa); %BFuww = 2.0337+(1.1285x%BFdxa). In regard to cross-calibrating the Hologic and Lunar DXA machines, DXA data were collected from 78 FLS subjects who were scanned on the same day with both the Hologic 4500 and Lunar LPX machines. The calibration equations are: Hologic %BF = 5.6397+0.7908×Lunar %BF; R^2^ = 0.98 and SE = 3.68; Hologic TBF = 3.0529+0.8439×Lunar TBF; R^2^ = 0.98 and SE = 1.47; Hologic FFM = 0.7917+1.0349×Lunar FFM; R^2^ = 0.94 and SE = 4.85. Bioelectrical impedance is proportional to total body water and to the length of the conductor or stature (stature*^2^*/resistance).

In adults, both SBP and DBP (mmHg) were recorded as the average of three readings from a mercury sphygmomanometer with participants in a seated position. Each reading was taken by rapidly inflating the arm cuff to the maximum level and deflating at a rate of 2mmHg per second, with 30 seconds between readings. Blood pressure was measured in children in accordance with the standards of the second National Heart, Lung, and Blood Institute Task Force on Blood Pressure Control in Children [31] and the update of that report by the National High Blood Pressure Education Program. [32]

Other covariates include self-reported physical activity (PA), alcohol use (ALC), and smoking status (SMK), as each has been shown by others to affect various aspects of cardiovascular or metabolic health. [17], [33]–[39] PA data are collected in the FLS using the Baecke Questionnaire of Habitual Physical Activity [40] and are recorded on a Likert scale. SMK is measured as the typical number of cigarettes smoked per day. ALC is defined as the typical number of alcoholic beverages consumed per day.

### Statistical Analyses

All analyses were performed using SAS/STAT software version 9.4 (SAS Institute Inc., Cary, NC, USA), while all figures were produced using the R computational software (version 2.12.2). Adult measurements were summarized with means, standard deviations, and 95% confidence intervals (adult age was also summarized with minimum and maximum values); note that childhood measurements were not numerically summarized due to the large number (33 possible) of measurements taken. A linear mixed-effect repeated measures ANOVA model was used to estimate childhood body size growth trajectories. The responses for this model are one of the seven repeated-measure childhood body size or composition measurements (BMI, WCHt, %BF, FFM, TBF, SBP and DBP). Fixed effects for this model include childhood age (rounded to the nearest half year – as per the study design – and categorized), one of the four continuous adult cardiac structure measurements (LVMI, RTW, IVSTs and IVSTd), and an interaction between the two. Note that particular cardiac structure measurements for participants (159 males and 186 females) with more than one echocardiographic visit were averaged into one representative value. A participant-level random effect was included to account for within-participant dependence, which was modeled using a first-order autoregressive structure. This model allowed for testing of ages of divergence of mean childhood body size, composition and blood pressure growth profiles based on “high” and “low” values of adult cardiac structure [21], where the body size, body composition and blood pressure means predicted at the first and third quartile of the adult cardiac structure level were compared at each age (from age 2 to 18 at 0.5-year increments). First and third quartiles of adult cardiac structure were used to represent healthy (low) and unhealthy (high) values, respectively, without being too extreme. To account for multiple comparisons (there are at most 33 such comparisons and as few as 8, since not all body size and composition measures are obtained at each age in all participants), the overall significance level of 0.05 was adjusted using the step-down approach to the Bonferroni correction. [41] Models were analyzed separately for each gender since boys and girls have different growth patterns. Comparisons were made in an unadjusted manner (as stated above), and are also made adjusting for three lifestyle measurements (PA, SMK, ALC), and were then adjusted for the adult body size, composition or blood pressure measurement in question. These adjustments were made to the linear mixed-effects model described above by including these adult body size, composition, blood pressure and lifestyle measurements as fixed effects. A sensitivity analysis excluding measurements taken in participants over 65 years was also conducted (though results are not reported). Inquiries on the data used in these analyses can be made to the corresponding author.

## Results

The adult echo-cardiographic measurements, as well as the adult body size, composition, and blood pressure measures are summarized in Table 1. Age, LVMI and RWT are similar between males and females, though the two IVST measures (systolic and diastolic) are on average smaller in women than in men. The two body size measurements (BMI and WCHt) are similar for men and women, while for the body composition measurements, males on average have lower %BF and TBF, and have higher FFM than females. As expected, both blood pressures are greater in males than in females. The three lifestyle measurements (ALC, SMK and PA) are similar between the sexes. For the results that follow, the sample size used for each unadjusted analysis is the minimum of the reported number of participants providing adult echocardiographic measurements in Table 1 (312 males, 385 females) and the number of participants providing at least one childhood measurement in Table 2. The sample size when adjusting for adult lifestyle measurements is the minimum of the number of participants with lifestyle measurements reported in Table 1 (ranges of 192–246 for males and 197–296 for females) and the number of participants with at least one childhood measurement reported in Table 2. The sample size when adjusting for adult body size, composition and blood pressure is the minimum of that reported for adult measurements in Table 1 (ranges of 143–329 for males and 165–411 for females) and the number of participants reported in Table 2.

**Table 1:** Data Summary of FLS Participant Measurements.

	Males				Females			
	N	Mean	SD	95% CI	N	Mean	SD	95% CI
Adult Age	312	38.7	19.86	20.0, 96.6	385	41.5	19.66	20.0–92.4
LVMI	312	29.6	9.04	28.7, 30.5	385	28.1	8.98	27.2, 28.9
RWT	312	0.29	0.060	0.28, 0.30	385	0.29	0.056	0.28, 0.30
IVSTs	312	0.80	0.190	0.78, 0.81	385	0.72	0.152	0.71, 0.74
IVSTd	312	1.04	0.269	1.02, 1.07	385	0.94	0.222	0.92, 0.96
BMI	330	27.0	4.78	26.5, 27.5	411	26.3	5.53	25.7, 26.8
WCHt	327	55.5	7.88	54.6, 56.3	411	55.7	8.39	54.9, 56.5
%BF	143	23.5	9.00	22.0, 25.0	165	34.6	8.75	33.2, 35.9
FFM	304	66.2	8.86	65.2, 67.2	397	46.5	6.70	45.9, 47.2
TBF	304	19.9	7.90	19.0, 20.8	397	25.3	9.65	24.3, 26.2
SBP	329	121.9	13.74	120.4, 123.4	411	116.1	18.71	114.3, 117.9
DBP	181	82.6	11.42	81.0, 84.3	189	77.8	14.14	75.7, 79.8
ALC	244	0.7	0.92	0.6, 0.9	296	0.3	0.56	0.3, 0.4
SMK	192	3.5	8.22	2.4, 4.7	197	3.2	6.34	2.3, 4.1
PA	246	2.6	0.41	2.5, 2.6	296	2.5	0.43	2.4, 2.5

Sample sizes (N), means, SDs, 95% CIs (minimum and maximum provided for Adult Age) for adulthood cardiac structure, body size and composition, and lifestyle measurements. Cardiac Structure includes LVMI, RWT, and IVST (both systolic and diastolic). Body size, composition and blood pressure measurements include BMI, WCHt, %BF, FFM, TBF, SBP and DBP. Lifestyle measurements include ALC, SMK and PA.

SD: standard deviation.

Min, Max: Minimum and Maximum.

CI: Confidence Interval.

LVMI: Left-ventricular mass index, g/m^2.7^.

RWT: Relative wall thickness, cm.

IVSTs: interventricular septum thickness – systolic, cm.

IVSTd: interventricular septum thickness – diastolic, cm.

BMI: Body mass index, kg/m^2^.

WCHt: Waist circumference divided by height, %.

%BF: Percentage body fat, %.

FFM: Fat-free mass, kg.

TBF: Total body fat, kg.

ALC: Number of servings of alcohol consumed per day.

SMK: Number of cigarettes smoked per day.

PA: Physical activity index (scale 1–5).

**Table 2:** Distribution of Number of Childhood Measurements.

	Males				
Measure	# of Subjects	Average # of Measurements	SD	Min	Max
BMI	305	19.9	9.98	1	33
WCHt	229	11.9	6.60	1	26
%BF	177	5.0	2.88	1	11
FFM	149	5.0	2.84	1	12
TBF	149	5.0	2.86	1	12
SBP	304	12.6	6.34	1	27
DBP	200	5.6	3.73	1	15

	Females				
Measure	# of Subjects	Average # of Measurements	SD	Min	Max
BMI	335	19.3	10.17	1	33
WCHt	251	11.0	7.20	1	27
%BF	181	4.7	2.88	1	11
FFM	140	4.9	2.97	1	11
TBF	140	4.9	2.97	1	11
SBP	332	12.0	6.09	1	24
DBP	193	4.9	3.49	1	16

Number of subjects with at least one childhood measurement for each of body size (BMI, WCHt), body composition (%BF, FFM, TBF), and blood pressure (SBP, DBP) for each gender. Average number of childhood measurements per participant, standard deviation (SD) and minimum (Min) and maximum (Max) are also reported.

### Left Ventricular Mass Index

**Figure 1:**
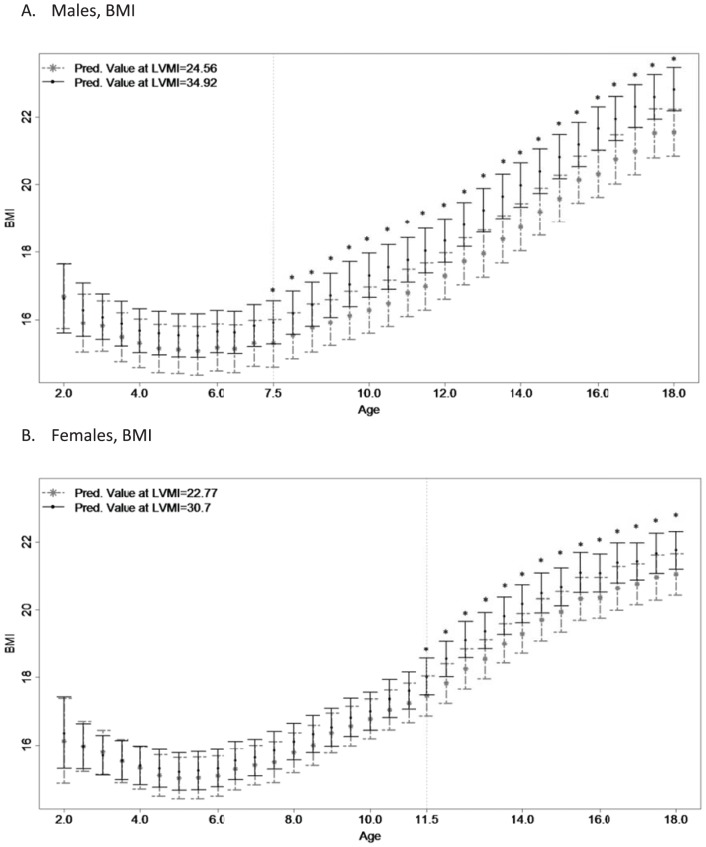
Childhood Body Mass Index Trajectories based upon Adulthood Left Ventricular Mass Index. *Figure 1* *Legend*: Childhood growth trajectories of body mass index (BMI) are provided for men's (N = 305; high: 34.92, low: 24.56) and women's (N = 335; high: 30.70, low: 22.77) left ventricular mass index (LVMI). Asterisks indicate significant results using Bonferroni-adjusted significance levels with the step-down approach.

The growth trajectories for BMI according to the first and third quartiles of adult LVMI become significantly different at age 7.5 in males (Figure 1A) and at age 11.5 in females (Figure 1B) and remain significant thereafter. In both males and females, participants with larger adult LVMI had larger childhood BMI than those adults with lower adult LVMI. After adjusting for adult lifestyle characteristics (Table 3), the age of divergence increases slightly to 8.0 in males, while the age of divergence for females remains 11.5. After adjusting for adult BMI (Table 3), no age of divergence in childhood BMI is detectable.

**Table 3:** Estimated “Age of Divergence” in Childhood Measurements.

		Males				Females	
		Unadujst	Adjusted Life	Adjusted Adult	Unadjust	Adjusted Life	Adjusted Adult
BMI	LVMI	7.5 years	8.0 years	None	11.5 years	11.5 years	None
	RWT	None			None		
	IVSTs	9.0 years	None		None		
	IVSTd	9.5 years	None		None		
					
WCHt	LVMI	8.0 years	8.0 years	None	None		
	RWT	None			None		
	IVSTs	None			None		
	IVSTd	None			None		
			
%BF		None			None		
FFM		None			None		
TBF		None			None		
SBP		None			None		
DBP		None			None		

These estimates are determined from sequential hypothesis tests of predicted childhood body size (BMI, WCHt), body composition (%BF, FFM, TBF) and blood pressure (SBP, DBP) compared at the first and third quartiles of adult cardiac structure (LVMI, RWT, IVSTs, IVSTd). These estimates are provided unadjusted for any other measurements, adjusted for adult lifestyle (Life) measurements (ALC, SMK and PA), and adjusted for the corresponding adult body size, body composition, or blood pressure measurement (Life/Adult). “None” implies that no hypotheses were rejected at any childhood age for that adult echocardiographic measurement.

**Figure 2:**
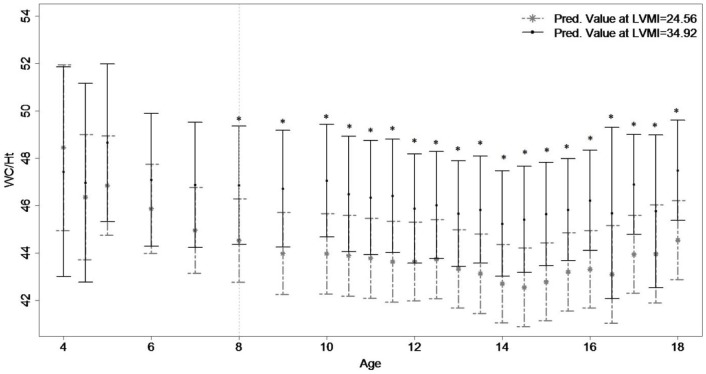
Childhood Waist Circumference Divided by Height Trajectories based upon Adulthood Left Ventricular Mass Index. *Figure 2* *Legend*: Childhood growth trajectories of waist circumference divided by height (WCHt) are provided for men's' (N = 229; high: 34.92, low: 24.56) and women's (N = 251; high: 30.70, low 22.77) left ventricular mass index (LVMI). Asterisks indicate significant results using Bonferroni-adjusted significance levels with the step-down approach.

The growth trajectories for WCHt according to adult LVMI (Figure 2) become significantly different at age 8 in males and generally remain significant thereafter (though the differences at age 16.5 and 17.5 are not significant). There was no significant divergence in WCHt growth in females based on LVMI. Participants with larger adult LVMI had larger childhood WCHt than those adults with lower adult LVMI. The age of divergence in childhood WCHt remained unchanged when adjusting for adult lifestyle measurements (Table 3), but disappeared when accounting for adult WCHt.

There were no significant differences in the childhood growth trends based on adult LVMI for the three body composition measurements (%BF, FFM, TBF) or the two blood pressures (SBP and DBP). Plots of all growth-trends based on adult LVMI are provided in Files S1, S2, S3, S4, S5, S6, and S7.

### Relative Wall Thickness

The growth trends for childhood body size, body composition and blood pressure were not significantly different between adults with high and low RWT in males or females. Plots of all growth-trends based on adult RWT levels are provided in Files S1, S2, S3, S4, S5, S6, and S7.

### Interventricular Septal Wall Thickness

**Figure 3:**
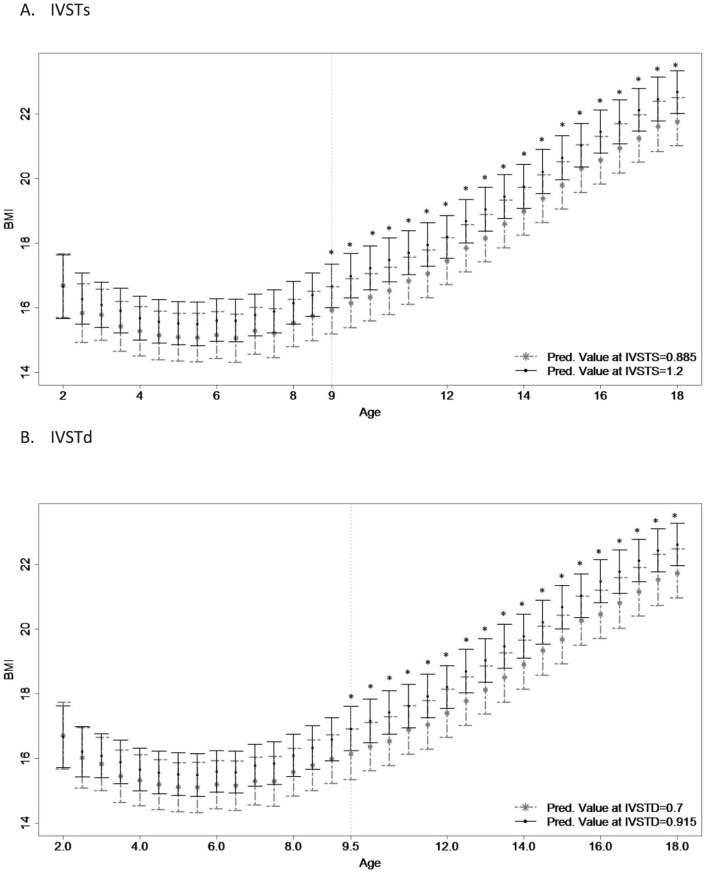
Childhood Body Mass Index Trajectories based upon Adulthood Interventricular Septal Thickness – Systolic and – Diastolic. *Figure 3* *Legend*: Childhood growth trajectories of body mass index (BMI) are provided for men's interventricular septal thickness – systolic (N = 305; IVSTs; high: 1.200, low: 0.885) and men's interventricular septal thickness – diastolic (N = 305; IVSTd; high: 0.915, low 0.700). Asterisks indicate significant results using Bonferroni-adjusted significance levels with the step-down approach.

In males, the growth trajectories for childhood BMI were significantly different according to adult IVSTs from ages 9 through 18 (Figure 3A). Adults with larger adult IVSTs had larger average childhood BMI than adults with lower IVSTs. These results were no longer significant when adjusted for adult lifestyle measurements or adult BMI. Growth trajectories for WCHt were significantly different at ages 9 and 10 but not thereafter, and thus do not constitute an age of divergence. None of the childhood body size, composition or blood pressure trends were significantly different in females based on adult IVSTs level.

In males, the childhood BMI growth trajectories were significantly different according to adult IVSTd between ages 9.5 to 18 (Figure 3B), where adults with larger IVSTd had larger childhood BMI than did adults with lower IVSTd. These differences were no longer significant when adjusted for adult lifestyle or adult BMI. Childhood %BF in males was significantly larger in adults with larger adult IVSTd than in adults with lower adult IVSTd at age 8, and childhood FFM was significantly higher in adults with larger IVSTd than in adults with lower IVSTd at ages 15 and 16. As no significant differences were observed after these ages, they did not constitute an age of divergence. None of the body size and composition trends were significantly different in females based on adult IVSTd level. Plots of all growth-trends based on adult IVSTs and IVSTd are provided in Files S1, S2, S3, S4, S5, S6, and S7.

## Discussion

Our methodological approach was to treat childhood body size, composition and blood pressure measurements as the repeated measure dependent outcome, and to treat adult cardiac structure as an independent variable. While this approach is the reverse of what would seem a more natural formulation with the adult measure as the dependent variable and the childhood measure as the independent variable, it allowed us to determine associations between the childhood and adult measures. Particularly, these results show that certain aspects of cardiac structure in adults are correlated with childhood body size, with the associations showing up in some cases before age 10. This seems to imply that some childhood characteristics – whether through childhood diet, behavior, activity level, or genetic predisposition – partially explain adult cardiac health. In boys, having larger BMI or waist circumference (indexed by height) in childhood is positively associated with developing abnormal LVMI and IVST in adulthood, while in girls, having larger BMI in childhood is positively associated with developing abnormal LVMI in adulthood.

Specifically, we found that certain childhood body size measurements, such as BMI and WCHt are associated with certain adulthood cardiac structure measurements. Using the unique longitudinal data of the Fels Longitudinal Study and its sub-sample of echocardiographic measurements, we were able to show that the childhood BMI for adults with high LVMI and IVST becomes significantly different from the childhood BMI for adults with low LVMI and IVST before puberty in boys. For girls, the age of BMI divergence occurred later than that of boys for LVMI, and was non-existent based upon IVST. WCHt also experienced an early divergence in boys based upon adult LVMI, but no significant divergence was observed in girls. There were no significant associations between childhood body size with RWT, and in general there were no significant divergences in childhood body composition or blood pressure based on adult cardiac structure.

The ages of divergence were mostly independent of adult lifestyle characteristics (ALC, SMK, and PA), and were no longer significant once adult body size and/or composition were accounted for. Similar results were seen in Sabo *et al*. (2012), who estimated age of divergences in childhood body size based on adult blood pressure.[21] While this phenomenon may seem to suggest that adult obesity or adiposity are more important in the relationship with cardiac structure than are childhood growth trends, it must be remembered that both childhood obesity and adiposity track into adulthood.[42] In addition, two research teams from the Bogalusa Heart Study also found – using echocardiographic measurements – that childhood BMI was positively associated with left ventricular structure, even after accounting for (young) adult body size.[22], [23] Regardless of the effects of adult body size and composition, it appears that the genesis of adult cardiac structure may be affected by body size in childhood, though earlier for boys than for girls.

Several studies have found that high blood pressure is one of the primary causes of LVH.[16], [17], [43] Rademacher *et al*. (2009) showed that childhood blood pressure and BMI exert independent influences on further cardiovascular risk, [10] while Malcolm *et al*. (1993) and de Simone *et al*. (1998) showed that the effect of body size on contemporaneous LVM was independent of both age and blood pressure. [12], [13] Schussheim *et al*. (2007) showed that patients with subnormal left ventricular shortening fraction (LVSF) have significantly higher diastolic blood pressure and greater BMI than patients matched for age and sex with normal LVSF. [44] de Simone also found associations between blood pressure and LVSF. [45] Interestingly, we did not find any associations between childhood blood pressure and adult cardiac structure. At least with respect to DBP, this lack of association may be due to the relatively low number of FLS participants providing measurements.

One limitation of our work is that this FLS subset only contains measurements on European-American participants. Therefore, generalizations of these findings to the entire US population or to other races should be avoided. Note that Toprak *et al*. did find that African Americans had a larger incidence of concentric left ventricular hypertrophy than did European-Americans, [22] but it is unclear if that implies that there are also racial disparities in the estimated ages of divergence observed here. The FLS sub-sample studied here also varied widely in age, from 20 years over 90 years. Though echocardiographic measurements were taken on these older individuals, a sensitivity analysis excluding measurements taken when participants were over 65 years did not change the result (results not shown). We also did not account for other measurements (such as diabetes status, HDL cholesterol, triglycerides and urinary albumin-creatinine ratio), though none of these measurements were significantly associated with adult cardiac structure in the Bogalusa study [22]. The predominant strength of this work is the large average number of repeated measurements per participant, which allowed us to estimate ages of divergence for each adult cardiac structure measurement based upon childhood body size, composition and blood pressure. Also unique was the coupling of adult cardiac structure with serial childhood measures.

## Supporting Information

File S1 **Childhood growth trajectories of body mass index (BMI) are provided for men's and women's left ventricular mass index (LVMI), relative wall thickness (RWT), interventricular septum thickness – systolic (IVSTs), andinterventricular septum thickness – diastolic (IVSTd).** Asterisks indicate significant results using Bonferroni-adjusted significance levels with the step-down approach.
(EPS)

File S2 **Childhood growth trajectories of waist circumference divided by height (WCHt) are provided for men's and women's left ventricular mass index (LVMI), relative wall thickness (RWT), interventricular septum thickness – systolic (IVSTs), andinterventricular septum thickness – diastolic (IVSTd).** Asterisks indicate significant results using Bonferroni-adjusted significance levels with the step-down approach.
(EPS)

File S3 **Childhood growth trajectories of percentage body fat (PBF) are provided for men's and women's left ventricular mass index (LVMI), relative wall thickness (RWT), interventricular septum thickness – systolic (IVSTs), andinterventricular septum thickness – diastolic (IVSTd).** Asterisks indicate significant results using Bonferroni-adjusted significance levels with the step-down approach.
(EPS)

File S4 **Childhood growth trajectories of fat free mass (FFM) are provided for men's and women's left ventricular mass index (LVMI), relative wall thickness (RWT), interventricular septum thickness – systolic (IVSTs), andinterventricular septum thickness – diastolic (IVSTd).** Asterisks indicate significant results using Bonferroni-adjusted significance levels with the step-down approach.
(EPS)

File S5 **Childhood growth trajectories of total body fat (TBF) are provided for men's and women's left ventricular mass index (LVMI), relative wall thickness (RWT), interventricular septum thickness – systolic (IVSTs), andinterventricular septum thickness – diastolic (IVSTd).** Asterisks indicate significant results using Bonferroni-adjusted significance levels with the step-down approach.
(EPS)

File S6 **Childhood growth trajectories of systolic blood pressure (SBP) are provided for men's and women's left ventricular mass index (LVMI), relative wall thickness (RWT), interventricular septum thickness – systolic (IVSTs), andinterventricular septum thickness – diastolic (IVSTd).** Asterisks indicate significant results using Bonferroni-adjusted significance levels with the step-down approach.
(EPS)

File S7 **Childhood growth trajectories of diastolic blood pressure (DBP) are provided for men's and women's left ventricular mass index (LVMI), relative wall thickness (RWT), interventricular septum thickness – systolic (IVSTs), andinterventricular septum thickness – diastolic (IVSTd).** Asterisks indicate significant results using Bonferroni-adjusted significance levels with the step-down approach.
(EPS)
